# Analysis of Adverse Events Following Phenobarbital Administration for Pediatric Patients Categorized by One-Year Age Increments Using the U.S. Food and Drug Administration Adverse Event Reporting System

**DOI:** 10.7759/cureus.56418

**Published:** 2024-03-18

**Authors:** Toru Ogura, Chihiro Shiraishi

**Affiliations:** 1 Clinical Research Support Center, Mie University Hospital, Tsu, JPN; 2 Department of Pharmacy, Mie University Hospital, Tsu, JPN

**Keywords:** cutoff value, database, large sample size, minimum p-value approach, occurrence tendency, seizure, wilcoxon–mann–whitney test

## Abstract

Background

Organ and body development greatly varies in pediatric patients from year to year. Therefore, the incidence of each adverse event following phenobarbital (PB) administration would vary with age. However, in clinical trials, increasing the sample size of pediatric patients in each age group has been challenging. Therefore, previous studies were conducted by dividing pediatric patients into three or four age groups based on the development stage. Although these results were useful in clinical settings, information on adverse events that occurred at one-year age increments in pediatric patients could further enhance treatment and care.

Objectives

This study investigated in one-year age increments the occurrence tendency of each adverse event following PB administration in pediatric patients.

Methods

This study used data obtained from the U.S. Food and Drug Administration Adverse Event Reporting System (FAERS). Two inclusion criteria were set: (1) treatment with PB between January 2004 and June 2023 and (2) age 0-15 years. Using the cutoff value obtained using the Wilcoxon-Mann-Whitney test by the minimum p-value approach, this study explored changes in the occurrence tendency of each adverse event in one-year age increments. At the minimum p-value of <0.05, the age corresponding to this p-value was determined as the cutoff value. Conversely, at the minimum p-value of ≥0.05, the cutoff value was considered nonexistent.

Results

This study investigated all types of adverse events and explored the cutoff value for each adverse event. We identified 34, 16, 15, nine, five, five, eight, three, and eight types of adverse events for the cutoff values of ≤3/>3, ≤4/>4, ≤5/>5, ≤6/>6, ≤7/>7, ≤8/>8, ≤9/>9, ≤10/>10, and ≤11/>11 years, respectively.

Conclusions

This study demonstrated that adverse events requiring attention in pediatric patients varied with age. The findings help in the improvement of treatment and care in the pediatric clinical settings.

## Introduction

Phenobarbital (PB) was approved as a treatment for epileptic seizures in 1912; it remains a first-line drug for treating neonatal seizures even more than 100 years after its approval [1,2]. Therefore, multiple studies have investigated PB administration in pediatric patients [3,4]. PB exerts its effect through the enhancement of GABA-ergic inhibition and reduction of glutamatergic excitation via inhibition of AMPA (alpha-amino-3-hydroxy-5-methyl-4-isoxazole propionic acid) receptors [5]. The types of adverse events following PB administration may differ between pediatric and adult patients, as previously reported [6]. In pediatric patients, adverse events classified as “more common” category include cognitive [7], psychogenic [7], sleep [8], and oral [9,10]. In all patients, the types of known adverse events following PB administration have been classified into three categories based on their incidence [6,11]: “more common,” “less common,” and “not known.” Confusion, somnolence, sweating, fatigue, dizziness, and vision blurred have been included under the “more common” category of adverse events, whereas muscle spasms, nausea, pyrexia, vomiting, coma, dyspnea, headache, and urine output decreased have been included under the “less common” category. Adverse events categorized as “not known” include skin lesion, diarrhea, cough, thoughts of killing oneself, muscular weakness, and changes in behavior. In study using the network meta-analysis, the frequency of adverse events following PB administration was reported in descending order of mood or behavioral change, cognitive disorder, and ataxia [12].

Several previous studies classified into groups based on the child development stage (newborn, infant, toddler, preschooler, school-aged child, and adolescent) and age [13]. For example, in some studies, the age groups created were 2-5 years, 6-12 years, and 13-18 years [14], thus involving a wide age range within each group. However, the development of the organs and bodies of pediatric patients varies greatly from year to year [15]. Therefore, the types of adverse events that are more likely to occur following PB administration would change with age. The availability of information in one-year age increments would be useful in clinical settings. However, increasing the sample size of pediatric patients in each age group in clinical trials is difficult. Systematic reviews and meta-analyses have shown that adverse events following PB administration can differ by age [16], but further details on such findings are not available.

Data registered in the U.S. Food and Drug Administration Adverse Event Reporting System (FAERS) [17] encompass volumes of adverse events reported worldwide since the first quarter of 2004 (2004Q1), including those on approximately 600,000 pediatric patients aged 0-15 years. The huge sample size obtained from the FAERS allowed us to study the occurrence tendency of each adverse event for pediatric patients in one-year age increments. However, calculating the incidence of each adverse event was impossible because the FAERS data included no reports with zero adverse events. Therefore, statistical analyses of the FAERS data in previous studies were distinguished from the original statistical analysis methods by adding “reporting” to the name of the methods, such as the reporting proportion (RP) [18,19] and reporting odds ratio [20].

## Materials and methods

Data source

This study used data obtained from the Adverse Event Reporting System (AERS) and FAERS database, which encompasses data registered between 2004Q1 and 2012Q3 and between 2012Q4 and 2023Q2, respectively. The AERS (aers_ascii_yyyyQq.zip) and FAERS (faers_ascii_yyyyQq.zip) data files were downloaded (yyyy and q present the year and quarter, respectively) on August 7, 2023. Differences were noted between the AERS and FAERS data, which were addressed based on the variable descriptions provided. Therefore, hereafter, references to the FAERS data include the AERS data. The FAERS data comprised seven files, of which the following were included for analysis: patient demographic and administrative information (DEMOyyQq.txt; yy presents the last two digits of the year), drug information (DRUGyyQq.txt), adverse event information (REACyyQq.txt), and drug therapy start and end dates (THERyyQq.txt). When new information is added to existing data in the FAERS, existing data are added to the database by incrementing the safety report version number {caseversion} rather than being overwritten. In this study, variable names used in the FAERS data were indicated using curly braces; the same applies hereinafter. Therefore, only the largest number of {caseversion} was used. However, similar judgments were possible from the number for identifying a case {CASE}, in spite of the {caseversion} not being provided in the AERS data. Data-handling, such as adjustment of the unit of age to years, the unit of weight to kilograms, and responding to unexpected inputs, was required when using {sex}, patient's age at event {age}, {weight}, and country of the reporter {reporter_country} for statistical analyses.

Approval from an institutional review board is not required, as the FAERS is an unlinkable anonymized database open to the public.

Study design

The following two inclusion criteria were set: (1) treatment with PB between 2004Q1 and 2023Q2 and (2) age 0-15 years. Between 2014Q3 and 2023Q2, PB could be searched on the FAERS by trade name using the variable of the product active ingredient {prod_ai}. Between 2004Q1 and 2014Q2, PB had to be searched by both trade and brand names using the variable of medical product {drugname} because prod_ai was not provided. The brand names included “Phenob,” “Donnatal,” “Gardenale,” “Luminal,” “Nobelbar,” and “Vegetamin.”

The exclusion criterion was patients who had never been administered PB before the occurrence of adverse events. These were determined using the date that the therapy was started (or re-started) for the drug {start_dt}, the date that the adverse event occurred or began {event_dt}, and the date that therapy with the drug was stopped {end_dt}. Cases wherein the {start_dt}, {event_dt}, and {end_dt} were only year, only year and month, or missing were noted. Because the sample size was smaller when cases with missing data for these three dates were excluded, only cases that could be reliably judged to be cases wherein PB was started following the occurrence of adverse events were excluded.

The endpoint was adverse event occurrence. This was provided as the preferred term {pt} level of medical terminology describing the event, using the Medical Dictionary for Regulatory Activities [20]. Although {pt} provided in the FAERS data are sometimes displayed in British English, this study was not rewritten out of respect for the originality of the FAERS data.

Statistical analyses

Continuous and categorical data were summarized as median (first and third quartiles) and frequency (RP), respectively, where RP = (number of patients with category of interest for target age) / (number of patients for target age) × 100. A scatterplot of the age and RP of each adverse event was constructed. To investigate the occurrence tendency of each adverse event at one-year age increments, the Wilcoxon-Mann-Whitney (WMW) test by the minimum p-value approach [21] was used to determine the cutoff value. The p-values of the WMW test for all potential cutoff values were calculated. When the minimum p-value was <0.05, the age corresponding to this p-value was determined as the cutoff value. Conversely, when the minimum p-value was ≥0.05, the cutoff value was considered nonexistent. If the RP was larger or smaller than others in only one or two age categories, it was considered coincidental; therefore, the potential cutoff values were set as ≤3/>3, ≤4/>4, ≤5/>5, ≤6/>6, ≤7/>7, ≤8/>8, ≤9/>9, ≤10/>10, and ≤11/>11 years. The two subgroups divided by the cutoff value both contained data for at least four age categories (the subgroup of ≤3 years included 0, 1, 2, and 3 years, and the subgroup of >11 years included 12, 13, 14, and 15 years). The software R version 4.1.3 (R Foundation for Statistical Computing, Vienna, Austria) was used for statistical analyses.

## Results

Patient background

**Figure 1:**
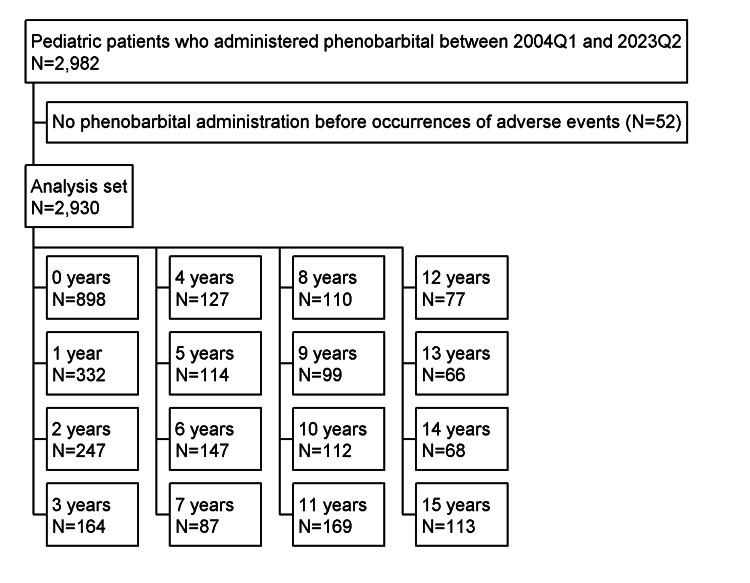
Flowchart of pediatric patients who were administered phenobarbital.

In total, we identified 2,982 pediatric patients who were administered PB between 2004Q1 and 2023Q2. After excluding 52 patients, eventually 2,930 patients were included in the analysis set. Figure 1 shows the breakdown of each age from the analysis set. The sample size for each age confirmed that it was sufficient. Table 1 summarizes the patient background.

**Table 1:** Summary of patient background Weight is summarized as median and first and third quartiles. Other data are summarized as frequency (reporting proportion). Q1, first quartile; Q3, third quartile; RP, reporting proportion

	0 years	1 year	2 years	3 years	4 years	5 years	6 years	7 years	8 years	9 years	10 years	11 years	12 years	13 years	14 years	15 years
	N=898	N=332	N=247	N=164	N=127	N=114	N=147	N=87	N=110	N=99	N=112	N=169	N=77	N=66	N=68	N=113
Sex																
Female, n (RP)	395 (44.0)	148 (44.6)	128 (51.8)	86 (52.4)	40 (31.5)	44 (38.6)	53 (36.1)	35 (40.2)	53 (48.2)	37 (37.4)	61 (54.5)	45 (26.6)	29 (37.7)	28 (42.4)	34 (50.0)	65 (57.5)
Male, n (RP)	456 (50.8)	168 (50.6)	108 (43.7)	73 (44.5)	86 (67.7)	69 (60.5)	89 (60.5)	44 (50.6)	57 (51.8)	61 (61.6)	50 (44.6)	124 (73.4)	48 (62.3)	36 (54.5)	33 (48.5)	48 (42.5)
Unknown, n (RP)	47 (5.2)	16 (4.8)	11 (4.5)	5 (3.0)	1 (0.8)	1 (0.9)	5 (3.4)	8 (9.2)	0 (0.0)	1 (1.0)	1 (0.9)	0 (0.0)	0 (0.0)	2 (3.0)	1 (1.5)	0 (0.0)
Weight, kg																
Median	4.5	9.6	12.0	12.5	15.0	18.2	16.0	22.0	25.0	29.0	27.9	38.3	38.0	32.0	45.0	36.5
Q1-Q3	2.6-7.0	7.7-10.0	9.9-13.7	10.0-16.0	13.8-17.8	16.0-22.1	14.0-22.7	19.0-25.2	20.4-31.4	21.9-37.0	17.0-28.9	30.0-38.3	28.7-45.8	21.3-32.9	40.0-61.5	28.8-46.0
Unknown, n (RP)	607 (67.6)	235 (70.8)	191 (77.3)	123 (75.0)	87 (68.5)	88 (77.2)	118 (80.3)	63 (72.4)	84 (76.4)	71 (71.7)	86 (76.8)	145 (85.8)	47 (61.0)	53 (80.3)	57 (83.8)	93 (82.3)
Country																
United States, n (RP)	375 (41.8)	156 (47.0)	112 (45.3)	61 (37.2)	53 (41.7)	44 (38.6)	63 (42.9)	26 (29.9)	21 (19.1)	33 (33.3)	34 (30.4)	29 (17.2)	28 (36.4)	20 (30.3)	24 (35.3)	42 (37.2)
Japan, n (RP)	91 (10.1)	31 (9.3)	23 (9.3)	21 (12.8)	14 (11.0)	11 (9.6)	16 (10.9)	11 (12.6)	10 (9.1)	10 (10.1)	12 (10.7)	17 (10.1)	9 (11.7)	17 (25.8)	9 (13.2)	19 (16.8)
Canada, n (RP)	68 (7.6)	14 (4.2)	0 (0.0)	5 (3.0)	2 (1.6)	0 (0.0)	1 (0.7)	11 (12.6)	9 (8.2)	12 (12.1)	5 (4.5)	78 (46.2)	3 (3.9)	1 (1.5)	1 (1.5)	1 (0.9)
Italy, n (RP)	44 (4.9)	25 (7.5)	10 (4.0)	11 (6.7)	6 (4.7)	20 (17.5)	2 (1.4)	2 (2.3)	21 (19.1)	6 (6.1)	14 (12.5)	15 (8.9)	1 (1.3)	14 (21.2)	3 (4.4)	11 (9.7)
Germany, n (RP)	49 (5.5)	24 (7.2)	2 (0.8)	10 (6.1)	4 (3.1)	1 (0.9)	12 (8.2)	0 (0.0)	5 (4.5)	2 (2.0)	0 (0.0)	2 (1.2)	12 (15.6)	0 (0.0)	1 (1.5)	0 (0.0)
France, n (RP)	46 (5.1)	8 (2.4)	20 (8.1)	6 (3.7)	7 (5.5)	0 (0.0)	1 (0.7)	2 (2.3)	0 (0.0)	0 (0.0)	5 (4.5)	2 (1.2)	2 (2.6)	0 (0.0)	2 (2.9)	2 (1.8)
Brazil, n (RP)	19 (2.1)	7 (2.1)	7 (2.8)	5 (3.0)	1 (0.8)	5 (4.4)	5 (3.4)	2 (2.3)	5 (4.5)	8 (8.1)	10 (8.9)	3 (1.8)	7 (9.1)	3 (4.5)	2 (2.9)	3 (2.7)
United Kingdom, n (RP)	22 (2.4)	16 (4.8)	2 (0.8)	2 (1.2)	2 (1.6)	2 (1.8)	1 (0.7)	1 (1.1)	0 (0.0)	9 (9.1)	2 (1.8)	8 (4.7)	1 (1.3)	0 (0.0)	4 (5.9)	12 (10.6)
India, n (RP)	17 (1.9)	1 (0.3)	5 (2.0)	2 (1.2)	6 (4.7)	2 (1.8)	1 (0.7)	6 (6.9)	17 (15.5)	2 (2.0)	4 (3.6)	2 (1.2)	5 (6.5)	2 (3.0)	7 (10.3)	1 (0.9)
China, n (RP)	20 (2.2)	3 (0.9)	10 (4.0)	4 (2.4)	9 (7.1)	0 (0.0)	0 (0.0)	0 (0.0)	1 (0.9)	3 (3.0)	1 (0.9)	1 (0.6)	1 (1.3)	1 (1.5)	3 (4.4)	4 (3.5)
Others, n (RP)	133 (14.8)	41 (12.3)	52 (21.1)	32 (19.5)	19 (15.0)	28 (24.6)	37 (25.2)	20 (23.0)	18 (16.4)	11 (11.1)	20 (17.9)	9 (5.3)	6 (7.8)	6 (9.1)	11 (16.2)	15 (13.3)
Unknown, n (RP)	14 (1.6)	6 (1.8)	4 (1.6)	5 (3.0)	4 (3.1)	1 (0.9)	8 (5.4)	6 (6.9)	3 (2.7)	3 (3.0)	5 (4.5)	3 (1.8)	2 (2.6)	2 (3.0)	1 (1.5)	3 (2.7)

Adverse events

In total, we identified 1,611 types of adverse events with one or more reports among patients aged 0-15 years. For all these adverse events, the WMW test by the minimum p-value approach was conducted. We identified 34, 16, 15, nine, five, five, eight, three, and eight types of adverse events for the cutoff values of ≤3/>3, ≤4/>4, ≤5/>5, ≤6/>6, ≤7/>7, ≤8/>8, ≤9/>9, ≤10/>10, and ≤11/>11 years, respectively.

**Figure 2:**
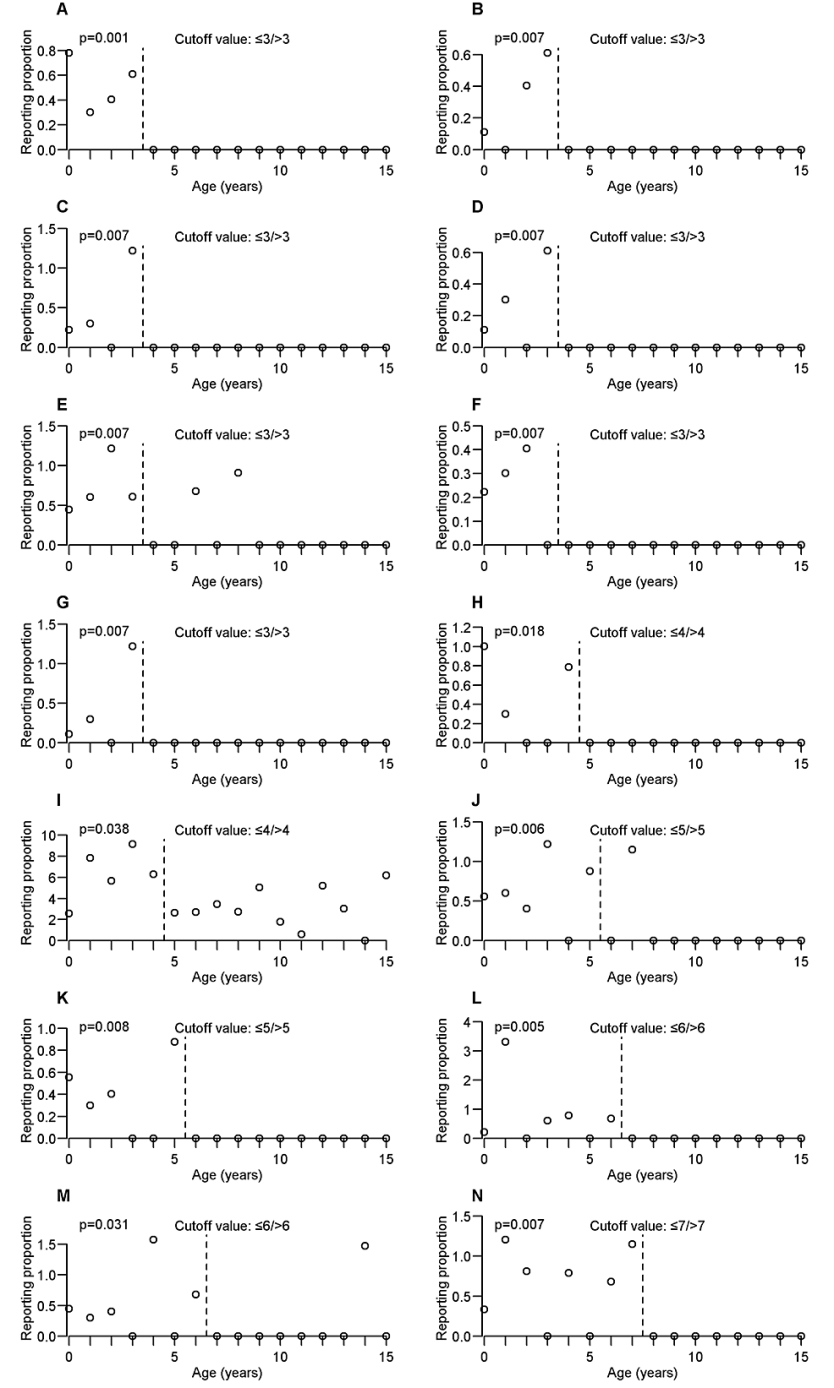
Cutoff value and scatterplot of age and reporting proportion of each adverse event. (A) Peritonitis, (B) cerebral haemorrhage, (C) haemorrhage intracranial, (D) hyperreflexia, (E) liver disorder, (F) pulmonary congestion, (G) skin lesion, (H) dysmorphism, (I) pneumonia, (J) cardiac failure, (K) hyperglycaemia, (L) hypertonia, (M) intellectual disability, and (N) upper respiratory tract infection.

**Figure 3:**
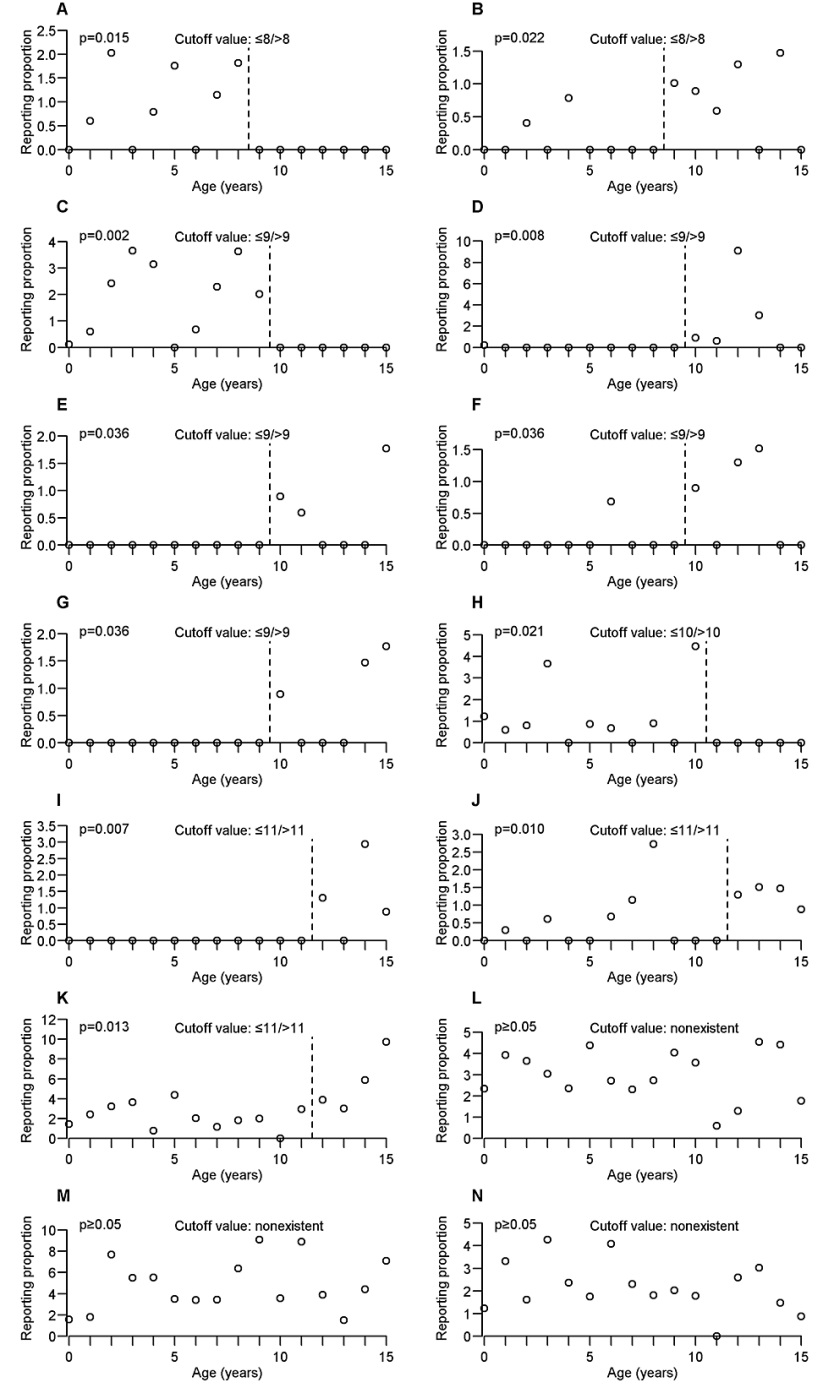
Cutoff value and scatterplot of age and reporting proportion of each adverse event (continued from Figure 2). (A) Generalized rash, (B) nausea, (C) Stevens-Johnson syndrome, (D) withdrawal syndrome, (E) decubitus ulcer, (F) drug withdrawal syndrome, (G) inflammation, (H) irritability, (I) implant site infection, (J) abnormal behaviour, (K) somnolence, (L) convulsion, (M) pyrexia, and (N) vomiting.

Figures 2, 3 show the cutoff value and scatterplot of age and RP of the adverse events as discussed in the Results and Discussion sections. However, although the cutoff values for convulsion, pyrexia, and vomiting were considered nonexistent, scatterplots were generated because they were discussed in the Results and Discussion sections. For the remaining types of adverse events that were determined to have the cutoff value, Appendix A summarizes the cutoff value and p-value obtained using the WMW test for each adverse event.

The occurrence tendency of each adverse event determined in this study was classified into three categories: (1) adverse events occasionally reported for ≤ cutoff value and almost never reported for > cutoff value, (2) adverse events almost never reported for ≤ cutoff value and occasionally reported for > cutoff value, and (3) adverse events reported from age 0 to 15 years, but with the occurrence tendency changed at the cutoff value.

Among the adverse events that fell under category 1, peritonitis (Figure 2A), cerebral hemorrhage (Figure 2B), hemorrhage intracranial (Figure 2C), hyperreflexia (Figure 2D), pulmonary congestion (Figure 2F), and skin lesion (Figure 2G) demonstrated a very distinctive occurrence tendency because they were occasionally reported in patients aged 0-3 years but not in patients aged 4-15 years. The occurrence tendency of these adverse events was completely differentiated at ≤3/>3 years. Furthermore, similar occurrence tendencies were observed for adverse events with other cutoff values, such as dysmorphism (Figure 2H) with the cutoff value of ≤4/>4 years, hyperglycemia (Figure 2K) with the cutoff value of ≤5/>5 years, hypertonia (Figure 2L) with the cutoff value of ≤6/>6 years, upper respiratory tract infection (Figure 2N) with the cutoff value of ≤7/>7 years, generalized rash (Figure 3A) with the cutoff value of ≤8/>8 years, Stevens-Johnson syndrome (Figure 3C) with the cutoff value of ≤9/>9 years, and irritability (Figure 3H) with the cutoff value of ≤10/>10 years. Some adverse events were not completely differentiated at the cutoff value but had similar characteristics. Liver disorder (Figure 2E) was occasionally reported in patients aged 0-3 years, seldom reported in patients aged 4-8 years, and not reported in patients aged 9-15 years. Similar occurrence tendencies were observed for cardiac failure (Figure 2J) and intellectual disability (Figure 2M).

Among the adverse events that fell under category 2, nausea (Figure 3B) with the cutoff value of ≤8/>8 years was almost never reported in patients aged 0-8 years, although it was occasionally reported in patients aged 9-15 years. Similar occurrence tendencies were observed for adverse events with other cutoff values, such as withdrawal syndrome (Figure 3D), decubitus ulcer (Figure 3E), drug withdrawal syndrome (Figure 3F), and inflammation (Figure 3G) with the cutoff value of ≤9/>9 years, and implant site infection (Figure 3I) with the cutoff value of ≤11/>11 years. Abnormal behavior (Figure 3J) with the cutoff value of 11/>11 years was occasionally reported in patients aged 0-11 years, but not in patients aged 0, 2, 4, 5, 9, 10, and 11 years. In contrast, abnormal behavior was occasionally reported at all ages in 12-15 years.

Among the adverse events that fell under category 3, pneumonia (Figure 2I) was occasionally reported in patients aged 0-4 years, but less frequently in patients aged 5-15 years than in those aged 0-4 years. Somnolence (Figure 3K) was occasionally reported in patients aged 0-11 years and even more frequently in patients aged 12-15 years.

Additionally, even for the adverse events for which the cutoff value was considered nonexistent, unique occurrence tendency characteristics were observed. Convulsion (Figure 3L), pyrexia (Figure 3M), and vomiting (Figure 3N) were occasionally reported in patients aged 0-15 years, although the cutoff value was considered nonexistent. Thus, these adverse events may need to be carefully monitored in patients aged 0-15 years.

Appendix B summarizes each adverse event of cognitive, psychogenic, sleep, and oral for which the cutoff value was considered nonexistent.

## Discussion

Previous clinical trials comparing newborns and infants following PB administration have revealed differences in the characteristics between the two development stages [3,4]. For pediatric patients, specifically for newborns, infants, toddlers, and preschoolers, organ and body development can make a big difference in one year. Previous studies provided sufficient information on adverse events in pediatric patients and have shown that adverse events following PB administration can differ by age [16]. However, they have not been investigated specifically at what age and which adverse events increase or decrease. Therefore, establishing the occurrence tendency of each adverse event at one-year age increments would be meaningful in clinical settings. Combining the results of previous studies with this study enabled the following interpretations to be established.

This study reported that liver disorder (Figure 2E) requires more attention at age ≤3 years. A previous study reported that PB-induced liver disorder was due to a hypersensitivity reaction [22]. Furthermore, the pharmacokinetic parameters (volume of distribution and clearance) of PB in newborns and infants were positively correlated with height and weight [3]. Therefore, the change in the occurrence tendency of liver disorder at the cutoff value may be related to the ongoing development of pediatric patients’ organs and bodies. For adverse events classified as category 1, the cutoff value in some cases was the school-aged children. The reason for this may be that the bodies of school-aged children greatly varied from year to year based on their age. Stevens-Johnson syndrome (Figure 3C) is an adverse event that requires caution following PB administration [23]. This study clarified that such caution should be especially exercised in patients aged ≤9 years. Previous studies have reported that drug withdrawal syndrome may occur following the discontinuation PB administration [24,25]. However, these reports did not provide information on age in pediatric patients. This study reports that withdrawal syndrome (Figure 3D) and drug withdrawal syndrome (Figure 3F) are seldom observed in patients aged 0-9 years but occasionally occur in patients aged 10-15 years. Furthermore, clinicians should be careful of withdrawal syndrome when stopping PB following long-term administration in pediatric patients. Decubitus ulcer (Figure 3E) and inflammation (Figure 3G) had similar occurrence tendencies as withdrawal syndrome and drug withdrawal syndrome. Presumably, these adverse events are more likely to occur with increasing PB administration period. Studies on long-term PB administration have reported different types of adverse events in comparison to those on short-term PB administration [26]. Somnolence (Figure 3K) is a commonly reported adverse event [6]. Although somnolence should be monitored at age 0-15 years, this study shows that the occurrence tendency may change at ≤11/>11 years. The FAERS data are frequently used to evaluate drug safety; however, such data can also be used to evaluate the efficacy of prophylactic drugs. Because PB is frequently administered to prevent convulsions [27], its efficacy may be evaluated based on the occurrence of convulsions in patients who receive the treatment. The variations in the efficacy of PB with age might not be large because convulsion (Figure 3L) was occasionally reported throughout patients aged 0-15 years, and the cutoff value for convulsion is nonexistent.

Adverse events of cognitive, psychogenic, sleep, and oral, which fall into the "more common" category in pediatric patients, were less frequently reported in the FAERS data. This may be because FAERS is a spontaneous report, there is a possibility that adverse events may not be reported even if they occur. Additionally, although it is difficult for newborns, infants, toddlers, and preschoolers to claim their own symptoms, adverse events due to subjective symptoms may be underestimated. Abnormal behavior (Figure 3J) and intellectual disability (Figure 2M) determined to have cutoff values are interpreted as follows. Although abnormal behavior should be monitored at age 0-15 years, the RPs are high, especially at age 12-15 years. Intellectual disability occasionally occurs in patients aged 0-6 years but is seldom observed in patients aged 7-15 years. Information on age, which requires special attention for these adverse events, has been added. Some adverse events such as gingival hypertrophy and oral disorder occasionally occur in patients aged 4-12 years but are not reported in patients aged 0-3 and 13-15 years. These results correspond to a previous study showing a high incidence of gingivitis in patients aged 6-11 years [28]. Because this study investigated in one-year age increments, it is also possible to confirm that these adverse events have this characteristic. A previous study reported that dental plaque appears to form more rapidly in patients aged 8-12 years than in adults [28]. Because there is a relationship between gingival overgrowth and plaque [29], appropriate plaque control is important for prevention and symptom improvement.

Other antiepileptic drugs on the market include levetiracetam [30], valproate [12], carbamazepine [12], and phenytoin [12]. Adverse events following levetiracetam administration included irritability, somnolence, dizziness, and asthenia [30]. Adverse events following valproate, carbamazepine, or phenytoin administration included somnolence, fatigue, headache, and dizziness [12]. As introduced in the Introduction, because somnolence, dizziness, and fatigue are classified into the “more common” category of adverse events after PB administration, the characteristics of adverse events between PB and other antiepileptic drugs are similar. This study shows that irritability and somnolence have the cutoff values. The occurrence tendency of each adverse event following other antiepileptic drug administration may also vary depending on age. This will be a topic for future research.

This study has some limitations. First, the FAERS data contained no cases with zero occurrences of each adverse event; thus, the incidence of each adverse event could not be calculated. Second, because the FAERS data were collected from spontaneous reports, they may contain bias. Third, the FAERS data contained numerous missing data. Finally, because the FAERS data did not provide laboratory values, the causal relationship between PB and adverse events could not be elucidated.

Despite these limitations, the study had the advantage of a large sample size that included numerous reports worldwide. This advantage has allowed us to study the occurrence tendency of each adverse event following PB administration for pediatric patients in one-year age increments, which is a strength of this study. This strength allowed us to confirm that the occurrence tendency of each adverse event following PB administration in pediatric patients varied in one-year age increments. However, the evidence level may be low because findings are based only on the FAERS data. Therefore, our findings need to be discussed in conjunction with the incidence of each adverse event reported in previous studies. Furthermore, our findings should be confirmed through further studies.

## Conclusions

The novelty of this study was to investigate pediatric patients in one-year age increments. This study demonstrates that the occurrence tendency of each adverse event following PB administration in pediatric patients varied in one-year age increments. Therefore, the various adverse events that require attention following PB administration vary with age. The results of this study interpreted with the incidence of each adverse event reported in previous studies provide information that would enhance treatment and care in pediatric clinical settings.

## Appendices

Appendix A

**Table 2:** Summary of each adverse event and cutoff value (not listed in Figures 2, 3). Data are summarized as frequency (reporting proportion). Cutoff, cutoff value calculated form the Wilcoxon-Mann-Whitney test by the minimum p-value approach; ≤3/>3, divided into ≤3 years and >3 years; ≤4/>4, divided into ≤4 years and >4 years; ≤5/>5, divided into ≤5 years and >5 years; ≤6/>6, divided into ≤6 years and >6 years; ≤7/>7, divided into ≤7 years and >7 years; ≤8/>8, divided into ≤8 years and >8 years; ≤9/>9, divided into ≤9 years and >9 years; ≤10/>10, divided into ≤10 years and >10 years; ≤11/>11, divided into ≤11 years and >11 years.

Adverse event name	0 years	1 year	2 years	3 years	4 years	5 years	6 years	7 years	8 years	9 years	10 years	11 years	12 years	13 years	14 years	15 years	Cutoff	p-Value
	N=898	N=332	N=247	N=164	N=127	N=114	N=147	N=87	N=110	N=99	N=112	N=169	N=77	N=66	N=68	N=113		
Cyanosis	1 (0.1)	13 (3.9)	2 (0.8)	1 (0.6)	0 (0.0)	0 (0.0)	0 (0.0)	0 (0.0)	0 (0.0)	0 (0.0)	0 (0.0)	0 (0.0)	0 (0.0)	1 (1.5)	0 (0.0)	0 (0.0)	≤3/>3	0.002
Cytomegalovirus infection	2 (0.2)	1 (0.3)	4 (1.6)	1 (0.6)	0 (0.0)	0 (0.0)	0 (0.0)	0 (0.0)	0 (0.0)	0 (0.0)	0 (0.0)	0 (0.0)	0 (0.0)	0 (0.0)	0 (0.0)	1 (0.9)	≤3/>3	0.002
Rhinovirus infection	6 (0.7)	5 (1.5)	2 (0.8)	0 (0.0)	0 (0.0)	0 (0.0)	0 (0.0)	0 (0.0)	0 (0.0)	0 (0.0)	0 (0.0)	0 (0.0)	0 (0.0)	0 (0.0)	0 (0.0)	0 (0.0)	≤3/>3	0.007
Wrong technique in drug usage process	2 (0.2)	2 (0.6)	0 (0.0)	1 (0.6)	0 (0.0)	0 (0.0)	0 (0.0)	0 (0.0)	0 (0.0)	0 (0.0)	0 (0.0)	0 (0.0)	0 (0.0)	0 (0.0)	0 (0.0)	0 (0.0)	≤3/>3	0.007
Tracheitis	1 (0.1)	0 (0.0)	1 (0.4)	1 (0.6)	0 (0.0)	0 (0.0)	0 (0.0)	0 (0.0)	0 (0.0)	0 (0.0)	0 (0.0)	0 (0.0)	0 (0.0)	0 (0.0)	0 (0.0)	0 (0.0)	≤3/>3	0.007
Tracheostomy	1 (0.1)	0 (0.0)	1 (0.4)	1 (0.6)	0 (0.0)	0 (0.0)	0 (0.0)	0 (0.0)	0 (0.0)	0 (0.0)	0 (0.0)	0 (0.0)	0 (0.0)	0 (0.0)	0 (0.0)	0 (0.0)	≤3/>3	0.007
Hypernatraemia	2 (0.2)	2 (0.6)	3 (1.2)	1 (0.6)	0 (0.0)	0 (0.0)	1 (0.7)	0 (0.0)	0 (0.0)	0 (0.0)	6 (5.4)	0 (0.0)	0 (0.0)	0 (0.0)	0 (0.0)	0 (0.0)	≤3/>3	0.008
Infantile spasms	17 (1.9)	7 (2.1)	2 (0.8)	0 (0.0)	0 (0.0)	0 (0.0)	0 (0.0)	0 (0.0)	0 (0.0)	1 (1.0)	0 (0.0)	0 (0.0)	0 (0.0)	0 (0.0)	0 (0.0)	0 (0.0)	≤3/>3	0.014
Pulmonary haemorrhage	7 (0.8)	1 (0.3)	1 (0.4)	1 (0.6)	0 (0.0)	0 (0.0)	0 (0.0)	0 (0.0)	0 (0.0)	0 (0.0)	0 (0.0)	0 (0.0)	1 (1.3)	0 (0.0)	0 (0.0)	1 (0.9)	≤3/>3	0.014
Hospitalisation	3 (0.3)	3 (0.9)	3 (1.2)	0 (0.0)	0 (0.0)	0 (0.0)	1 (0.7)	0 (0.0)	0 (0.0)	0 (0.0)	0 (0.0)	0 (0.0)	0 (0.0)	0 (0.0)	0 (0.0)	0 (0.0)	≤3/>3	0.014
Brain oedema	3 (0.3)	0 (0.0)	2 (0.8)	1 (0.6)	0 (0.0)	0 (0.0)	0 (0.0)	0 (0.0)	0 (0.0)	0 (0.0)	0 (0.0)	1 (0.6)	0 (0.0)	0 (0.0)	0 (0.0)	0 (0.0)	≤3/>3	0.014
Movement disorder	1 (0.1)	1 (0.3)	2 (0.8)	1 (0.6)	0 (0.0)	0 (0.0)	0 (0.0)	1 (1.1)	0 (0.0)	0 (0.0)	0 (0.0)	0 (0.0)	0 (0.0)	0 (0.0)	0 (0.0)	1 (0.9)	≤3/>3	0.014
Death	15 (1.7)	13 (3.9)	21 (8.5)	6 (3.7)	1 (0.8)	0 (0.0)	1 (0.7)	1 (1.1)	2 (1.8)	4 (4.0)	1 (0.9)	2 (1.2)	2 (2.6)	0 (0.0)	0 (0.0)	0 (0.0)	≤3/>3	0.017
Disease recurrence	11 (1.2)	1 (0.3)	1 (0.4)	0 (0.0)	0 (0.0)	0 (0.0)	0 (0.0)	0 (0.0)	0 (0.0)	0 (0.0)	1 (0.9)	0 (0.0)	0 (0.0)	0 (0.0)	0 (0.0)	0 (0.0)	≤3/>3	0.020
Vascular device infection	1 (0.1)	0 (0.0)	1 (0.4)	1 (0.6)	0 (0.0)	0 (0.0)	0 (0.0)	0 (0.0)	0 (0.0)	0 (0.0)	0 (0.0)	1 (0.6)	0 (0.0)	0 (0.0)	0 (0.0)	0 (0.0)	≤3/>3	0.020
Respiratory tract infection	4 (0.4)	1 (0.3)	1 (0.4)	3 (1.8)	0 (0.0)	0 (0.0)	2 (1.4)	0 (0.0)	0 (0.0)	0 (0.0)	2 (1.8)	0 (0.0)	0 (0.0)	0 (0.0)	0 (0.0)	1 (0.9)	≤3/>3	0.027
Abdominal distension	8 (0.9)	0 (0.0)	1 (0.4)	1 (0.6)	0 (0.0)	0 (0.0)	0 (0.0)	0 (0.0)	0 (0.0)	0 (0.0)	0 (0.0)	0 (0.0)	0 (0.0)	0 (0.0)	1 (1.5)	0 (0.0)	≤3/>3	0.027
Maternal exposure during pregnancy	5 (0.6)	1 (0.3)	1 (0.4)	0 (0.0)	0 (0.0)	0 (0.0)	0 (0.0)	0 (0.0)	1 (0.9)	0 (0.0)	0 (0.0)	0 (0.0)	0 (0.0)	0 (0.0)	0 (0.0)	0 (0.0)	≤3/>3	0.027
Pleural effusion	1 (0.1)	1 (0.3)	1 (0.4)	2 (1.2)	0 (0.0)	0 (0.0)	1 (0.7)	0 (0.0)	1 (0.9)	0 (0.0)	0 (0.0)	1 (0.6)	0 (0.0)	0 (0.0)	0 (0.0)	0 (0.0)	≤3/>3	0.027
Productive cough	5 (0.6)	0 (0.0)	1 (0.4)	1 (0.6)	0 (0.0)	0 (0.0)	0 (0.0)	0 (0.0)	0 (0.0)	0 (0.0)	0 (0.0)	0 (0.0)	0 (0.0)	0 (0.0)	0 (0.0)	1 (0.9)	≤3/>3	0.027
Myocarditis	1 (0.1)	0 (0.0)	3 (1.2)	1 (0.6)	0 (0.0)	0 (0.0)	0 (0.0)	0 (0.0)	0 (0.0)	0 (0.0)	0 (0.0)	0 (0.0)	0 (0.0)	0 (0.0)	1 (1.5)	0 (0.0)	≤3/>3	0.027
Cerebral palsy	2 (0.2)	1 (0.3)	1 (0.4)	0 (0.0)	0 (0.0)	0 (0.0)	0 (0.0)	0 (0.0)	0 (0.0)	0 (0.0)	0 (0.0)	0 (0.0)	0 (0.0)	0 (0.0)	1 (1.5)	0 (0.0)	≤3/>3	0.027
Brain death	1 (0.1)	1 (0.3)	1 (0.4)	0 (0.0)	0 (0.0)	0 (0.0)	0 (0.0)	0 (0.0)	0 (0.0)	0 (0.0)	0 (0.0)	0 (0.0)	0 (0.0)	1 (1.5)	0 (0.0)	0 (0.0)	≤3/>3	0.027
Lung disorder	1 (0.1)	1 (0.3)	1 (0.4)	0 (0.0)	0 (0.0)	0 (0.0)	0 (0.0)	0 (0.0)	0 (0.0)	0 (0.0)	1 (0.9)	0 (0.0)	0 (0.0)	0 (0.0)	0 (0.0)	0 (0.0)	≤3/>3	0.027
Myoclonic epilepsy	8 (0.9)	6 (1.8)	0 (0.0)	3 (1.8)	0 (0.0)	0 (0.0)	0 (0.0)	0 (0.0)	0 (0.0)	0 (0.0)	1 (0.9)	0 (0.0)	1 (1.3)	0 (0.0)	0 (0.0)	1 (0.9)	≤3/>3	0.030
Apnoea	12 (1.3)	2 (0.6)	1 (0.4)	0 (0.0)	0 (0.0)	0 (0.0)	0 (0.0)	0 (0.0)	0 (0.0)	1 (1.0)	0 (0.0)	1 (0.6)	0 (0.0)	0 (0.0)	0 (0.0)	0 (0.0)	≤3/>3	0.039
Gastrooesophageal reflux disease	6 (0.7)	1 (0.3)	0 (0.0)	2 (1.2)	0 (0.0)	0 (0.0)	0 (0.0)	0 (0.0)	0 (0.0)	1 (1.0)	0 (0.0)	1 (0.6)	0 (0.0)	0 (0.0)	0 (0.0)	0 (0.0)	≤3/>3	0.039
Rhinorrhoea	3 (0.3)	1 (0.3)	2 (0.8)	1 (0.6)	1 (0.8)	0 (0.0)	0 (0.0)	0 (0.0)	0 (0.0)	0 (0.0)	0 (0.0)	1 (0.6)	0 (0.0)	0 (0.0)	0 (0.0)	0 (0.0)	≤4/>4	0.001
Multiple organ dysfunction syndrome	8 (0.9)	1 (0.3)	1 (0.4)	2 (1.2)	1 (0.8)	0 (0.0)	0 (0.0)	1 (1.1)	0 (0.0)	0 (0.0)	0 (0.0)	0 (0.0)	0 (0.0)	0 (0.0)	0 (0.0)	0 (0.0)	≤4/>4	0.001
Respiratory syncytial virus infection	2 (0.2)	1 (0.3)	1 (0.4)	0 (0.0)	2 (1.6)	0 (0.0)	0 (0.0)	1 (1.1)	0 (0.0)	0 (0.0)	0 (0.0)	0 (0.0)	0 (0.0)	0 (0.0)	0 (0.0)	0 (0.0)	≤4/>4	0.010
Acute respiratory failure	5 (0.6)	2 (0.6)	2 (0.8)	2 (1.2)	1 (0.8)	0 (0.0)	0 (0.0)	0 (0.0)	0 (0.0)	0 (0.0)	1 (0.9)	0 (0.0)	2 (2.6)	0 (0.0)	0 (0.0)	0 (0.0)	≤4/>4	0.013
Medication error	1 (0.1)	2 (0.6)	0 (0.0)	1 (0.6)	1 (0.8)	0 (0.0)	0 (0.0)	2 (2.3)	0 (0.0)	0 (0.0)	0 (0.0)	0 (0.0)	0 (0.0)	0 (0.0)	0 (0.0)	0 (0.0)	≤4/>4	0.013
Altered state of consciousness	2 (0.2)	1 (0.3)	0 (0.0)	1 (0.6)	1 (0.8)	0 (0.0)	0 (0.0)	0 (0.0)	1 (0.9)	0 (0.0)	0 (0.0)	0 (0.0)	0 (0.0)	0 (0.0)	0 (0.0)	0 (0.0)	≤4/>4	0.013
Anticonvulsant drug level decreased	9 (1.0)	11 (3.3)	0 (0.0)	0 (0.0)	1 (0.8)	0 (0.0)	0 (0.0)	0 (0.0)	0 (0.0)	0 (0.0)	0 (0.0)	1 (0.6)	0 (0.0)	0 (0.0)	0 (0.0)	0 (0.0)	≤4/>4	0.018
Magnetic resonance imaging head abnormal	4 (0.4)	1 (0.3)	0 (0.0)	0 (0.0)	1 (0.8)	0 (0.0)	0 (0.0)	0 (0.0)	0 (0.0)	0 (0.0)	0 (0.0)	0 (0.0)	0 (0.0)	0 (0.0)	0 (0.0)	0 (0.0)	≤4/>4	0.018
Hepatocellular injury	0 (0.0)	0 (0.0)	1 (0.4)	1 (0.6)	2 (1.6)	0 (0.0)	0 (0.0)	0 (0.0)	0 (0.0)	0 (0.0)	0 (0.0)	0 (0.0)	0 (0.0)	0 (0.0)	0 (0.0)	0 (0.0)	≤4/>4	0.018
Neutrophil count decreased	1 (0.1)	1 (0.3)	0 (0.0)	0 (0.0)	2 (1.6)	0 (0.0)	0 (0.0)	0 (0.0)	0 (0.0)	0 (0.0)	0 (0.0)	0 (0.0)	0 (0.0)	0 (0.0)	0 (0.0)	0 (0.0)	≤4/>4	0.018
Speech disorder	0 (0.0)	0 (0.0)	1 (0.4)	1 (0.6)	2 (1.6)	0 (0.0)	0 (0.0)	0 (0.0)	0 (0.0)	0 (0.0)	0 (0.0)	0 (0.0)	0 (0.0)	0 (0.0)	0 (0.0)	0 (0.0)	≤4/>4	0.018
Lip disorder	1 (0.1)	0 (0.0)	1 (0.4)	0 (0.0)	1 (0.8)	0 (0.0)	0 (0.0)	0 (0.0)	0 (0.0)	0 (0.0)	0 (0.0)	0 (0.0)	0 (0.0)	0 (0.0)	0 (0.0)	0 (0.0)	≤4/>4	0.018
Drug level increased	1 (0.1)	0 (0.0)	0 (0.0)	0 (0.0)	0 (0.0)	1 (0.9)	1 (0.7)	1 (1.1)	0 (0.0)	0 (0.0)	1 (0.9)	1 (0.6)	0 (0.0)	1 (1.5)	2 (2.9)	2 (1.8)	≤4/>4	0.029
Thrombosis	2 (0.2)	0 (0.0)	0 (0.0)	3 (1.8)	1 (0.8)	0 (0.0)	0 (0.0)	0 (0.0)	0 (0.0)	0 (0.0)	0 (0.0)	1 (0.6)	0 (0.0)	0 (0.0)	0 (0.0)	0 (0.0)	≤4/>4	0.033
Influenza	1 (0.1)	2 (0.6)	1 (0.4)	2 (1.2)	1 (0.8)	2 (1.8)	0 (0.0)	0 (0.0)	0 (0.0)	0 (0.0)	0 (0.0)	0 (0.0)	0 (0.0)	1 (1.5)	0 (0.0)	0 (0.0)	≤5/>5	0.001
Brain operation	3 (0.3)	2 (0.6)	1 (0.4)	1 (0.6)	0 (0.0)	1 (0.9)	0 (0.0)	0 (0.0)	0 (0.0)	0 (0.0)	0 (0.0)	0 (0.0)	0 (0.0)	0 (0.0)	0 (0.0)	0 (0.0)	≤5/>5	0.001
Developmental delay	8 (0.9)	1 (0.3)	0 (0.0)	0 (0.0)	1 (0.8)	1 (0.9)	0 (0.0)	0 (0.0)	0 (0.0)	0 (0.0)	0 (0.0)	0 (0.0)	0 (0.0)	0 (0.0)	0 (0.0)	0 (0.0)	≤5/>5	0.008
Hypophagia	1 (0.1)	2 (0.6)	0 (0.0)	2 (1.2)	0 (0.0)	1 (0.9)	0 (0.0)	0 (0.0)	0 (0.0)	0 (0.0)	0 (0.0)	0 (0.0)	0 (0.0)	0 (0.0)	0 (0.0)	0 (0.0)	≤5/>5	0.008
Surgery	1 (0.1)	0 (0.0)	2 (0.8)	1 (0.6)	0 (0.0)	1 (0.9)	0 (0.0)	0 (0.0)	0 (0.0)	0 (0.0)	0 (0.0)	1 (0.6)	0 (0.0)	0 (0.0)	0 (0.0)	0 (0.0)	≤5/>5	0.015
Tachycardia	11 (1.2)	9 (2.7)	14 (5.7)	0 (0.0)	6 (4.7)	4 (3.5)	0 (0.0)	0 (0.0)	0 (0.0)	0 (0.0)	1 (0.9)	1 (0.6)	2 (2.6)	0 (0.0)	0 (0.0)	2 (1.8)	≤5/>5	0.018
Accidental overdose	5 (0.6)	0 (0.0)	8 (3.2)	1 (0.6)	0 (0.0)	1 (0.9)	0 (0.0)	0 (0.0)	0 (0.0)	0 (0.0)	0 (0.0)	0 (0.0)	0 (0.0)	0 (0.0)	2 (2.9)	0 (0.0)	≤5/>5	0.029
Hypoglycaemia	5 (0.6)	0 (0.0)	0 (0.0)	1 (0.6)	0 (0.0)	1 (0.9)	0 (0.0)	0 (0.0)	0 (0.0)	0 (0.0)	0 (0.0)	0 (0.0)	0 (0.0)	0 (0.0)	0 (0.0)	0 (0.0)	≤5/>5	0.036
Enterovirus infection	1 (0.1)	3 (0.9)	0 (0.0)	0 (0.0)	0 (0.0)	2 (1.8)	0 (0.0)	0 (0.0)	0 (0.0)	0 (0.0)	0 (0.0)	0 (0.0)	0 (0.0)	0 (0.0)	0 (0.0)	0 (0.0)	≤5/>5	0.036
Disorientation	1 (0.1)	0 (0.0)	0 (0.0)	2 (1.2)	0 (0.0)	2 (1.8)	0 (0.0)	0 (0.0)	0 (0.0)	0 (0.0)	0 (0.0)	0 (0.0)	0 (0.0)	0 (0.0)	0 (0.0)	0 (0.0)	≤5/>5	0.036
General physical health deterioration	3 (0.3)	0 (0.0)	0 (0.0)	1 (0.6)	0 (0.0)	1 (0.9)	0 (0.0)	0 (0.0)	0 (0.0)	0 (0.0)	0 (0.0)	0 (0.0)	0 (0.0)	0 (0.0)	0 (0.0)	0 (0.0)	≤5/>5	0.036
Kawasaki's disease	0 (0.0)	2 (0.6)	2 (0.8)	0 (0.0)	0 (0.0)	1 (0.9)	0 (0.0)	0 (0.0)	0 (0.0)	0 (0.0)	0 (0.0)	0 (0.0)	0 (0.0)	0 (0.0)	0 (0.0)	0 (0.0)	≤5/>5	0.036
Rash morbilliform	1 (0.1)	0 (0.0)	3 (1.2)	0 (0.0)	0 (0.0)	1 (0.9)	0 (0.0)	0 (0.0)	0 (0.0)	0 (0.0)	0 (0.0)	0 (0.0)	0 (0.0)	0 (0.0)	0 (0.0)	0 (0.0)	≤5/>5	0.036
Seizure cluster	2 (0.2)	3 (0.9)	2 (0.8)	1 (0.6)	1 (0.8)	1 (0.9)	2 (1.4)	0 (0.0)	0 (0.0)	0 (0.0)	0 (0.0)	0 (0.0)	1 (1.3)	0 (0.0)	0 (0.0)	0 (0.0)	≤6/>6	0.002
Flushing	7 (0.8)	6 (1.8)	0 (0.0)	1 (0.6)	1 (0.8)	0 (0.0)	3 (2.0)	0 (0.0)	0 (0.0)	0 (0.0)	0 (0.0)	0 (0.0)	0 (0.0)	0 (0.0)	0 (0.0)	0 (0.0)	≤6/>6	0.005
Haematochezia	4 (0.4)	1 (0.3)	1 (0.4)	0 (0.0)	0 (0.0)	0 (0.0)	1 (0.7)	0 (0.0)	0 (0.0)	0 (0.0)	0 (0.0)	0 (0.0)	0 (0.0)	0 (0.0)	0 (0.0)	0 (0.0)	≤6/>6	0.019
Lethargy	5 (0.6)	5 (1.5)	0 (0.0)	2 (1.2)	1 (0.8)	0 (0.0)	0 (0.0)	4 (4.6)	2 (1.8)	1 (1.0)	2 (1.8)	0 (0.0)	1 (1.3)	4 (6.1)	3 (4.4)	1 (0.9)	≤6/>6	0.024
Hyperammonaemia	6 (0.7)	6 (1.8)	0 (0.0)	0 (0.0)	1 (0.8)	6 (5.3)	13 (8.8)	0 (0.0)	0 (0.0)	0 (0.0)	0 (0.0)	6 (3.6)	0 (0.0)	0 (0.0)	0 (0.0)	0 (0.0)	≤6/>6	0.027
Rhabdomyolysis	6 (0.7)	5 (1.5)	0 (0.0)	1 (0.6)	0 (0.0)	1 (0.9)	1 (0.7)	0 (0.0)	0 (0.0)	0 (0.0)	0 (0.0)	0 (0.0)	0 (0.0)	0 (0.0)	0 (0.0)	5 (4.4)	≤6/>6	0.035
Erythema	1 (0.1)	1 (0.3)	3 (1.2)	0 (0.0)	0 (0.0)	1 (0.9)	2 (1.4)	0 (0.0)	0 (0.0)	0 (0.0)	0 (0.0)	5 (3.0)	0 (0.0)	0 (0.0)	0 (0.0)	0 (0.0)	≤6/>6	0.035
Drug dose omission	21 (2.3)	20 (6.0)	5 (2.0)	6 (3.7)	5 (3.9)	4 (3.5)	3 (2.0)	2 (2.3)	0 (0.0)	0 (0.0)	1 (0.9)	0 (0.0)	1 (1.3)	0 (0.0)	0 (0.0)	0 (0.0)	≤7/>7	<0.001
Hepatic enzyme increased	4 (0.4)	2 (0.6)	4 (1.6)	2 (1.2)	1 (0.8)	0 (0.0)	7 (4.8)	3 (3.4)	0 (0.0)	0 (0.0)	1 (0.9)	1 (0.6)	0 (0.0)	0 (0.0)	0 (0.0)	0 (0.0)	≤7/>7	0.011
Hypophosphataemia	4 (0.4)	0 (0.0)	0 (0.0)	1 (0.6)	1 (0.8)	0 (0.0)	1 (0.7)	1 (1.1)	0 (0.0)	0 (0.0)	0 (0.0)	0 (0.0)	0 (0.0)	0 (0.0)	0 (0.0)	0 (0.0)	≤7/>7	0.026
Hemiparesis	1 (0.1)	1 (0.3)	0 (0.0)	1 (0.6)	0 (0.0)	2 (1.8)	0 (0.0)	1 (1.1)	0 (0.0)	0 (0.0)	0 (0.0)	0 (0.0)	0 (0.0)	0 (0.0)	0 (0.0)	0 (0.0)	≤7/>7	0.026
Blindness	0 (0.0)	1 (0.3)	0 (0.0)	3 (1.8)	1 (0.8)	0 (0.0)	0 (0.0)	1 (1.1)	1 (0.9)	0 (0.0)	0 (0.0)	0 (0.0)	0 (0.0)	0 (0.0)	0 (0.0)	0 (0.0)	≤8/>8	0.034
No adverse event	19 (2.1)	5 (1.5)	2 (0.8)	1 (0.6)	0 (0.0)	0 (0.0)	1 (0.7)	1 (1.1)	2 (1.8)	0 (0.0)	0 (0.0)	0 (0.0)	0 (0.0)	1 (1.5)	0 (0.0)	0 (0.0)	≤8/>8	0.034
Hypoxia	5 (0.6)	1 (0.3)	1 (0.4)	1 (0.6)	0 (0.0)	1 (0.9)	1 (0.7)	0 (0.0)	2 (1.8)	0 (0.0)	0 (0.0)	0 (0.0)	0 (0.0)	0 (0.0)	0 (0.0)	1 (0.9)	≤8/>8	0.049
Anticonvulsant drug level increased	1 (0.1)	1 (0.3)	0 (0.0)	1 (0.6)	1 (0.8)	1 (0.9)	0 (0.0)	1 (1.1)	0 (0.0)	1 (1.0)	0 (0.0)	0 (0.0)	0 (0.0)	0 (0.0)	0 (0.0)	0 (0.0)	≤9/>9	0.018
Muscle spasms	6 (0.7)	1 (0.3)	1 (0.4)	1 (0.6)	3 (2.4)	0 (0.0)	1 (0.7)	1 (1.1)	1 (0.9)	1 (1.0)	0 (0.0)	1 (0.6)	0 (0.0)	0 (0.0)	0 (0.0)	1 (0.9)	≤9/>9	0.039
Anaemia	8 (0.9)	4 (1.2)	2 (0.8)	1 (0.6)	2 (1.6)	2 (1.8)	0 (0.0)	0 (0.0)	2 (1.8)	1 (1.0)	0 (0.0)	0 (0.0)	1 (1.3)	0 (0.0)	0 (0.0)	0 (0.0)	≤9/>9	0.045
Respiratory depression	12 (1.3)	3 (0.9)	0 (0.0)	3 (1.8)	1 (0.8)	0 (0.0)	0 (0.0)	0 (0.0)	0 (0.0)	1 (1.0)	0 (0.0)	68 (40.2)	1 (1.3)	3 (4.5)	4 (5.9)	0 (0.0)	≤10/>10	0.043
Hypokalaemia	3 (0.3)	2 (0.6)	1 (0.4)	0 (0.0)	0 (0.0)	2 (1.8)	1 (0.7)	1 (1.1)	0 (0.0)	0 (0.0)	7 (6.2)	0 (0.0)	0 (0.0)	0 (0.0)	0 (0.0)	0 (0.0)	≤10/>10	0.048
Aggression	0 (0.0)	0 (0.0)	1 (0.4)	0 (0.0)	1 (0.8)	1 (0.9)	0 (0.0)	0 (0.0)	0 (0.0)	0 (0.0)	0 (0.0)	0 (0.0)	2 (2.6)	1 (1.5)	1 (1.5)	1 (0.9)	≤11/>11	0.001
Respiratory rate decreased	0 (0.0)	0 (0.0)	0 (0.0)	1 (0.6)	0 (0.0)	0 (0.0)	0 (0.0)	0 (0.0)	0 (0.0)	0 (0.0)	0 (0.0)	0 (0.0)	0 (0.0)	1 (1.5)	1 (1.5)	1 (0.9)	≤11/>11	0.007
Depressed level of consciousness	8 (0.9)	0 (0.0)	0 (0.0)	1 (0.6)	1 (0.8)	0 (0.0)	0 (0.0)	2 (2.3)	3 (2.7)	1 (1.0)	0 (0.0)	0 (0.0)	7 (9.1)	1 (1.5)	2 (2.9)	1 (0.9)	≤11/>11	0.023
Eosinophilia	2 (0.2)	1 (0.3)	4 (1.6)	2 (1.2)	0 (0.0)	0 (0.0)	1 (0.7)	1 (1.1)	0 (0.0)	3 (3.0)	10 (8.9)	1 (0.6)	0 (0.0)	0 (0.0)	0 (0.0)	0 (0.0)	≤11/>11	0.034
Eye movement disorder	5 (0.6)	12 (3.6)	0 (0.0)	0 (0.0)	0 (0.0)	0 (0.0)	0 (0.0)	0 (0.0)	0 (0.0)	0 (0.0)	0 (0.0)	0 (0.0)	1 (1.3)	1 (1.5)	0 (0.0)	1 (0.9)	≤11/>11	0.039

Appendix B 

**Table 3:** Summary of each adverse event (cognitive, psychogenic, sleep, and oral) without cutoff value. Data are summarized as frequency (reporting proportion). Cutoff, cutoff value calculated form the Wilcoxon-Mann-Whitney test by the minimum p-value approach; -, cutoff value was considered nonexistent.

Adverse event name	0 years	1 year	2 years	3 years	4 years	5 years	6 years	7 years	8 years	9 years	10 years	11 years	12 years	13 years	14 years	15 years	Cutoff	p-value
	N=898	N=332	N=247	N=164	N=127	N=114	N=147	N=87	N=110	N=99	N=112	N=169	N=77	N=66	N=68	N=113		
Cognitive																		
Cognitive disorder	4 (0.4)	1 (0.3)	1 (0.4)	1 (0.6)	2 (1.6)	0 (0.0)	4 (2.7)	2 (2.3)	0 (0.0)	0 (0.0)	2 (1.8)	1 (0.6)	0 (0.0)	0 (0.0)	0 (0.0)	1 (0.9)	-	≥0.05
Focal dyscognitive seizures	0 (0.0)	1 (0.3)	0 (0.0)	0 (0.0)	1 (0.8)	0 (0.0)	0 (0.0)	0 (0.0)	0 (0.0)	0 (0.0)	0 (0.0)	0 (0.0)	0 (0.0)	2 (3.0)	0 (0.0)	0 (0.0)	-	≥0.05
Behaviour disorder	3 (0.3)	0 (0.0)	0 (0.0)	1 (0.6)	2 (1.6)	2 (1.8)	0 (0.0)	0 (0.0)	0 (0.0)	0 (0.0)	2 (1.8)	1 (0.6)	0 (0.0)	0 (0.0)	0 (0.0)	0 (0.0)	-	≥0.05
Impulsive behaviour	0 (0.0)	0 (0.0)	0 (0.0)	0 (0.0)	0 (0.0)	0 (0.0)	0 (0.0)	0 (0.0)	0 (0.0)	0 (0.0)	0 (0.0)	0 (0.0)	1 (1.3)	0 (0.0)	0 (0.0)	1 (0.9)	-	≥0.05
Suicidal behaviour	0 (0.0)	0 (0.0)	0 (0.0)	0 (0.0)	0 (0.0)	0 (0.0)	0 (0.0)	0 (0.0)	0 (0.0)	0 (0.0)	0 (0.0)	0 (0.0)	0 (0.0)	0 (0.0)	2 (2.9)	0 (0.0)	-	≥0.05
Disturbance in social behaviour	1 (0.1)	0 (0.0)	0 (0.0)	0 (0.0)	0 (0.0)	0 (0.0)	0 (0.0)	0 (0.0)	0 (0.0)	0 (0.0)	0 (0.0)	0 (0.0)	0 (0.0)	0 (0.0)	0 (0.0)	0 (0.0)	-	≥0.05
Neonatal behavioural syndrome	1 (0.1)	0 (0.0)	0 (0.0)	0 (0.0)	0 (0.0)	0 (0.0)	0 (0.0)	0 (0.0)	0 (0.0)	0 (0.0)	0 (0.0)	0 (0.0)	0 (0.0)	0 (0.0)	0 (0.0)	0 (0.0)	-	≥0.05
Self injurious behaviour	0 (0.0)	0 (0.0)	0 (0.0)	0 (0.0)	0 (0.0)	0 (0.0)	0 (0.0)	0 (0.0)	0 (0.0)	0 (0.0)	0 (0.0)	0 (0.0)	0 (0.0)	0 (0.0)	1 (1.5)	0 (0.0)	-	≥0.05
Learning disability	1 (0.1)	0 (0.0)	0 (0.0)	0 (0.0)	0 (0.0)	0 (0.0)	0 (0.0)	0 (0.0)	0 (0.0)	0 (0.0)	2 (1.8)	0 (0.0)	0 (0.0)	0 (0.0)	0 (0.0)	0 (0.0)	-	≥0.05
Learning disorder	1 (0.1)	0 (0.0)	0 (0.0)	0 (0.0)	0 (0.0)	0 (0.0)	0 (0.0)	0 (0.0)	0 (0.0)	0 (0.0)	0 (0.0)	0 (0.0)	1 (1.3)	0 (0.0)	0 (0.0)	0 (0.0)	-	≥0.05
Disturbance in attention	2 (0.2)	0 (0.0)	0 (0.0)	1 (0.6)	1 (0.8)	0 (0.0)	0 (0.0)	0 (0.0)	1 (0.9)	0 (0.0)	0 (0.0)	0 (0.0)	0 (0.0)	0 (0.0)	0 (0.0)	0 (0.0)	-	≥0.05
Psychogenic																		
Psychomotor hyperactivity	0 (0.0)	0 (0.0)	0 (0.0)	3 (1.8)	1 (0.8)	4 (3.5)	1 (0.7)	0 (0.0)	0 (0.0)	1 (1.0)	0 (0.0)	0 (0.0)	1 (1.3)	0 (0.0)	0 (0.0)	0 (0.0)	-	≥0.05
Paroxysmal sympathetic hyperactivity	0 (0.0)	0 (0.0)	0 (0.0)	0 (0.0)	0 (0.0)	0 (0.0)	3 (2.0)	0 (0.0)	0 (0.0)	0 (0.0)	0 (0.0)	0 (0.0)	0 (0.0)	0 (0.0)	0 (0.0)	0 (0.0)	-	≥0.05
Psychomotor skills impaired	4 (0.4)	0 (0.0)	0 (0.0)	0 (0.0)	0 (0.0)	0 (0.0)	1 (0.7)	0 (0.0)	0 (0.0)	0 (0.0)	0 (0.0)	0 (0.0)	0 (0.0)	0 (0.0)	0 (0.0)	0 (0.0)	-	≥0.05
Psychomotor retardation	2 (0.2)	0 (0.0)	0 (0.0)	0 (0.0)	0 (0.0)	0 (0.0)	1 (0.7)	0 (0.0)	0 (0.0)	0 (0.0)	0 (0.0)	0 (0.0)	0 (0.0)	0 (0.0)	0 (0.0)	0 (0.0)	-	≥0.05
Psychomotor disadaptation syndrome	0 (0.0)	1 (0.3)	0 (0.0)	0 (0.0)	0 (0.0)	0 (0.0)	0 (0.0)	0 (0.0)	0 (0.0)	0 (0.0)	0 (0.0)	0 (0.0)	0 (0.0)	0 (0.0)	0 (0.0)	0 (0.0)	-	≥0.05
Rebound psychosis	1 (0.1)	0 (0.0)	0 (0.0)	0 (0.0)	0 (0.0)	0 (0.0)	0 (0.0)	0 (0.0)	0 (0.0)	0 (0.0)	0 (0.0)	0 (0.0)	0 (0.0)	0 (0.0)	0 (0.0)	0 (0.0)	-	≥0.05
Substance-induced psychotic disorder	0 (0.0)	0 (0.0)	0 (0.0)	0 (0.0)	0 (0.0)	0 (0.0)	0 (0.0)	0 (0.0)	0 (0.0)	1 (1.0)	0 (0.0)	0 (0.0)	0 (0.0)	0 (0.0)	0 (0.0)	0 (0.0)	-	≥0.05
Mental retardation	1 (0.1)	0 (0.0)	0 (0.0)	0 (0.0)	0 (0.0)	0 (0.0)	0 (0.0)	0 (0.0)	0 (0.0)	1 (1.0)	0 (0.0)	0 (0.0)	0 (0.0)	0 (0.0)	2 (2.9)	3 (2.7)	-	≥0.05
Mental status changes	4 (0.4)	0 (0.0)	0 (0.0)	1 (0.6)	0 (0.0)	0 (0.0)	0 (0.0)	1 (1.1)	0 (0.0)	0 (0.0)	0 (0.0)	0 (0.0)	0 (0.0)	0 (0.0)	0 (0.0)	1 (0.9)	-	≥0.05
Mental disorder	1 (0.1)	0 (0.0)	0 (0.0)	1 (0.6)	0 (0.0)	0 (0.0)	0 (0.0)	0 (0.0)	0 (0.0)	0 (0.0)	0 (0.0)	0 (0.0)	0 (0.0)	0 (0.0)	0 (0.0)	0 (0.0)	-	≥0.05
Mental impairment	0 (0.0)	0 (0.0)	0 (0.0)	0 (0.0)	0 (0.0)	0 (0.0)	0 (0.0)	0 (0.0)	0 (0.0)	0 (0.0)	0 (0.0)	0 (0.0)	0 (0.0)	0 (0.0)	1 (1.5)	0 (0.0)	-	≥0.05
Neurodevelopmental disorder	0 (0.0)	0 (0.0)	2 (0.8)	0 (0.0)	0 (0.0)	0 (0.0)	0 (0.0)	0 (0.0)	0 (0.0)	0 (0.0)	0 (0.0)	0 (0.0)	0 (0.0)	0 (0.0)	0 (0.0)	0 (0.0)	-	≥0.05
Sleep																		
Sleep disorder	4 (0.4)	1 (0.3)	0 (0.0)	0 (0.0)	0 (0.0)	0 (0.0)	0 (0.0)	0 (0.0)	0 (0.0)	0 (0.0)	2 (1.8)	0 (0.0)	0 (0.0)	0 (0.0)	0 (0.0)	1 (0.9)	-	≥0.05
Circadian rhythm sleep disorder	0 (0.0)	0 (0.0)	0 (0.0)	0 (0.0)	0 (0.0)	2 (1.8)	0 (0.0)	0 (0.0)	0 (0.0)	0 (0.0)	0 (0.0)	0 (0.0)	0 (0.0)	0 (0.0)	0 (0.0)	0 (0.0)	-	≥0.05
Obstructive sleep apnoea syndrome	0 (0.0)	0 (0.0)	0 (0.0)	0 (0.0)	0 (0.0)	1 (0.9)	0 (0.0)	0 (0.0)	0 (0.0)	0 (0.0)	0 (0.0)	0 (0.0)	1 (1.3)	0 (0.0)	0 (0.0)	0 (0.0)	-	≥0.05
Sleep deficit	0 (0.0)	0 (0.0)	0 (0.0)	0 (0.0)	0 (0.0)	0 (0.0)	0 (0.0)	0 (0.0)	0 (0.0)	0 (0.0)	0 (0.0)	1 (0.6)	0 (0.0)	0 (0.0)	0 (0.0)	1 (0.9)	-	≥0.05
Poor quality sleep	1 (0.1)	0 (0.0)	0 (0.0)	0 (0.0)	0 (0.0)	0 (0.0)	0 (0.0)	0 (0.0)	0 (0.0)	0 (0.0)	0 (0.0)	0 (0.0)	0 (0.0)	0 (0.0)	0 (0.0)	0 (0.0)	-	≥0.05
Sleep apnoea syndrome	0 (0.0)	0 (0.0)	1 (0.4)	0 (0.0)	0 (0.0)	0 (0.0)	0 (0.0)	0 (0.0)	0 (0.0)	0 (0.0)	0 (0.0)	0 (0.0)	0 (0.0)	0 (0.0)	0 (0.0)	0 (0.0)	-	≥0.05
Sleep terror	0 (0.0)	0 (0.0)	1 (0.4)	0 (0.0)	0 (0.0)	0 (0.0)	0 (0.0)	0 (0.0)	0 (0.0)	0 (0.0)	0 (0.0)	0 (0.0)	0 (0.0)	0 (0.0)	0 (0.0)	0 (0.0)	-	≥0.05
Oral																		
Gingival hypertrophy	0 (0.0)	0 (0.0)	0 (0.0)	0 (0.0)	1 (0.8)	0 (0.0)	1 (0.7)	1 (1.1)	0 (0.0)	0 (0.0)	6 (5.4)	0 (0.0)	1 (1.3)	0 (0.0)	0 (0.0)	0 (0.0)	-	≥0.05
Gingival ulceration	0 (0.0)	0 (0.0)	0 (0.0)	0 (0.0)	0 (0.0)	0 (0.0)	0 (0.0)	0 (0.0)	0 (0.0)	0 (0.0)	2 (1.8)	0 (0.0)	0 (0.0)	0 (0.0)	0 (0.0)	0 (0.0)	-	≥0.05
Gingival swelling	0 (0.0)	0 (0.0)	0 (0.0)	0 (0.0)	0 (0.0)	0 (0.0)	0 (0.0)	0 (0.0)	0 (0.0)	0 (0.0)	0 (0.0)	0 (0.0)	1 (1.3)	0 (0.0)	0 (0.0)	0 (0.0)	-	≥0.05
Oral disorder	0 (0.0)	0 (0.0)	0 (0.0)	0 (0.0)	0 (0.0)	0 (0.0)	1 (0.7)	0 (0.0)	1 (0.9)	0 (0.0)	2 (1.8)	0 (0.0)	1 (1.3)	0 (0.0)	0 (0.0)	0 (0.0)	-	≥0.05
Oral candidiasis	2 (0.2)	0 (0.0)	0 (0.0)	0 (0.0)	0 (0.0)	0 (0.0)	0 (0.0)	0 (0.0)	0 (0.0)	0 (0.0)	0 (0.0)	0 (0.0)	0 (0.0)	0 (0.0)	0 (0.0)	0 (0.0)	-	≥0.05
Oral discharge	0 (0.0)	0 (0.0)	0 (0.0)	0 (0.0)	1 (0.8)	0 (0.0)	0 (0.0)	0 (0.0)	0 (0.0)	0 (0.0)	0 (0.0)	0 (0.0)	0 (0.0)	0 (0.0)	0 (0.0)	0 (0.0)	-	≥0.05
Oral fungal infection	0 (0.0)	0 (0.0)	0 (0.0)	0 (0.0)	1 (0.8)	0 (0.0)	0 (0.0)	0 (0.0)	0 (0.0)	0 (0.0)	0 (0.0)	0 (0.0)	0 (0.0)	0 (0.0)	0 (0.0)	0 (0.0)	-	≥0.05
Oral herpes	0 (0.0)	0 (0.0)	0 (0.0)	0 (0.0)	0 (0.0)	0 (0.0)	0 (0.0)	0 (0.0)	0 (0.0)	0 (0.0)	0 (0.0)	0 (0.0)	0 (0.0)	0 (0.0)	1 (1.5)	0 (0.0)	-	≥0.05
Tooth disorder	2 (0.2)	0 (0.0)	0 (0.0)	1 (0.6)	0 (0.0)	0 (0.0)	0 (0.0)	0 (0.0)	1 (0.9)	0 (0.0)	0 (0.0)	0 (0.0)	0 (0.0)	0 (0.0)	0 (0.0)	0 (0.0)	-	≥0.05
Tooth loss	0 (0.0)	0 (0.0)	0 (0.0)	1 (0.6)	0 (0.0)	0 (0.0)	0 (0.0)	0 (0.0)	2 (1.8)	0 (0.0)	0 (0.0)	0 (0.0)	1 (1.3)	0 (0.0)	0 (0.0)	0 (0.0)	-	≥0.05
Tooth development disorder	0 (0.0)	0 (0.0)	0 (0.0)	0 (0.0)	1 (0.8)	0 (0.0)	0 (0.0)	0 (0.0)	0 (0.0)	0 (0.0)	0 (0.0)	0 (0.0)	0 (0.0)	0 (0.0)	0 (0.0)	0 (0.0)	-	≥0.05
